# Exploring the Cooccurrence Patterns of Multiple Sets of Genomic Intervals

**DOI:** 10.1155/2013/617545

**Published:** 2013-05-28

**Authors:** Hao Wu, Zhaohui S. Qin

**Affiliations:** ^1^Department of Biostatistics and Bioinformatics, Emory University, Atlanta, GA, USA; ^2^Department of Biomedical Informatics, Emory University School of Medicine, Atlanta, GA, USA; ^3^Center for Comprehensive Informative, Emory University, Atlanta, GA, USA

## Abstract

*Background*. Exploring the spatial relationship of different genomic features has been of great interest since the early days of genomic research. The relationship sometimes provides useful information for understanding certain biological processes. Recent advances in high-throughput technologies such as ChIP-seq produce large amount of data in the form of genomic intervals. Most of the existing methods for assessing spatial relationships among the intervals are designed for pairwise comparison and cannot be easily scaled up. *Results*. We present a statistical method and software tool to characterize the cooccurrence patterns of multiple sets of genomic intervals. The occurrences of genomic intervals are described by a simple finite mixture model, where each component represents a distinct cooccurrence pattern. The model parameters are estimated via an EM algorithm and can be viewed as sufficient statistics of the cooccurrence patterns. Simulation and real data results show that the model can accurately capture the patterns and provide biologically meaningful results. The method is implemented in a freely available R package giClust. *Conclusions*. The method and the software provide a convenient way for biologists to explore the cooccurrence patterns among a relatively large number of sets of genomic intervals.

## 1. Introduction

Exploring the spatial relationships of different genomic features has been of great interest since the early days of genomic research. The relationships often provide important information for certain biological processes. One famous example is that people detected CpG islands (CGI) from the DNA sequence as short, CG rich genomic regions and then found that they significantly overlap gene promoters [1]. The CGI/promoter overlaps shed light on the function of DNA methylation on gene expressions.

 In modern functional genomics research, one major goal is to understand the regulatory mechanism of gene expression. The transcriptional process involves the combinatory effects of different DNA-binding proteins and histone modifications. To decipher the complex process, an important first step is to detect the protein binding or histone modification sites and then explore the spatial relationships among them. The spatial relationships provide evidence for interactions among various regulatory elements. For example, if the binding sites of two proteins significantly overlap, it is likely that they interact. Recent advances in high-throughput technologies such as ChIP-seq [2] make the genome-wide profiling of proteins binding or histone modification an easy task. It is now common for a biologist to map the binding sites for a few proteins and then compare them with each other or with some public data. Since the protein binding or histone modification sites are represented as genomic intervals, such task requires the comparison of multiple sets of genomic intervals. Hereafter a set of genomic intervals will be referred to as a “track.”

 Although good tools for comparing tracks are immediately needed, there are only a few existing methods, and most of them are designed for pairwise comparison. The easiest way to compare two tracks is to compute their overlaps and then represent them by a Venn diagram [3]. This method, however, does not evaluate the statistical significance of the overlaps. Several more statistically rigorous methods are recently proposed to evaluate the overlap or “closeness” of two tracks. Favorov et al. developed an R package called “GenometriCorr” to evaluate the spatial correlation between two tracks [4]. They implemented several test procedures to measure the “closeness” of the two tracks and report *P* values. Chikina and Troyanskaya proposed a similar distance based method for comparing two tracks [5]. One advantage of their method is that the relationship can be evaluated within a user-defined genomic regions (termed as “domain set” in the paper). All the methods discussed above are designed for comparing two tracks. Because these methods are based on pairwise overlaps or distances, they cannot be easily scaled up for comparing multiple tracks. To that end, a generalized linear model based approach was proposed to explore the dependence of one track on several others [6]. It first converts each track into a binary vector based on genome wide occurrence; then the occurrence of one track is modeled as a function of the occurrence of other tracks through a log-linear model. One drawback of such an approach is that it only measures the marginal dependence. For example, if track A significantly overlaps track B only in a small proportion of the genome, the marginal dependence will be small and likely to be overlooked. A newly developed method “ChromHMM” was proposed to discover the genome-wide chromatin state [7]. It first splits the genome into equal sized bins and then applies a hidden Markov model (HMM) with certain number of states to segment the genome according to the combinations of chromatin modifications. In this approach, the emission probabilities of the HMM are based on overlapping patterns of multiple chromatin modifications. However the method itself is not specifically designed for evaluating relationships of multiple sets of genomic regions. A newly developed method jMOSAiCS [8] implements a joint model for multiple sets of ChIP-seq data. Its main goal is to improve peak calling and cannot be directly applied to assess the spatial correlations among peak lists from different ChIP-seq data.

In this work, we aim to develop an intuitive tool for comparing multiple tracks. There are different types of spatial relationships. Consider the pairwise comparison, the most straightforward relationship is overlapping. In addition, two tracks could be close to each other but do not overlap. A biological example for the “closeness” relationship is differentially methylated regions (DMRs) and CGI. It was reported that DMRs are at the CGI “shores” [9], meaning that the two tracks are close but not overlapping. Moreover, two tracks could “exclude” each other. In this work, we focus only on the overlapping relationship and present a statistical method to characterize the cooccurrence patterns of multiple tracks. The patterns are described by a finite mixture model. An EM algorithm [10] is devised to infer the model parameters. Given multiple tracks and a set of user provided genomic regions, the method clusters the regions into certain number of groups according to the cooccurrence patterns among the input tracks. Moreover, the model parameters “sufficiently” summarize the cooccurrence patterns among input tracks. The sufficiency means that any joint or conditional occurrence probabilities in a subset of the inputs can be calculated from the parameters alone. For example, questions like “what percent of the sites bound by both protein A and B are also bound by protein C” can be readily answered without the raw data.

## 2. Methods

 Assume that there are *D* input tracks and one wants to explore their cooccurrence patterns over a set of *N* genomic intervals (referred to as “regions of interest” hereafter). The regions of interest are specified by the user. An example is the promoter regions of all known genes. The first step in analysis is to count the overlaps of input tracks and the regions of interest. Let *Y*_*id*_ indicate that the *i*th region of interest overlaps some interval from *d*th track (=1) or not (=0), *i* = 1,…, *N*, *d* = 1,…, *D*. The *Y*_*id*_ matrix is the input data for the following procedures.

### 2.1. A Finite Mixture Model for the Cooccurrence Patterns

The cooccurrence pattern of the input tracks is described by a finite mixture model. Each mixture component is represented by a product of Bernoulli distributions. Assume that the regions of interest are from a mixture of *K* clusters. In each cluster, the input tracks exhibit distinct cooccurrence patterns. Let *Z*_*i*_ denote the cluster indicator for the *i*th region of interest. Assume that the prior probability for a region of interest being in the *k*th cluster is *π*_*k*_; for example, Pr(*Z*_*i*_ = *k*) = *π*_*k*_, ∑_*k*=1_^*K*^ *π*_*k*_ = 1. For a region of interest in cluster *k*, let the probability of intervals from track *d* occurring in this regions be *q*_*kd*_; in other words, *P*(*Y*_*id*_ = 1 | *Z*_*i*_ = *k*) = *q*_*kd*_. Define **Q** ≡ {*q*_*kd*_; *k* = 1,…, *K*; *d* = 1,…, *D*}, a *K* × *D* matrix. This matrix characterizes the cooccurrence patterns of input tracks over the regions of interest. We further assume that within a cluster, the occurrences of input tracks are independent; for example, *P*(*Y*_*il*_ = 1,  *Y*_*il*′_ = 1 | *Z*_*i*_ = *k*) = *P*(*Y*_*il*_ = 1 | *Z*_*i*_ = *k*)*P*(*Y*_*il*′_ = 1 | *Z*_*i*_ = *k*) for all *l*, *l*′.

### 2.2. Parameter Estimation

 Under the proposed model, *Y*_*id*_s are observed data, *Z*_*i*_s are missing indicator variables, and *q*_*kd*_ and *π*_*k*_s are model parameters. *K* represent the dimension of the model, which can be either specified by the user or estimated from data. Given *K*, the model parameters can be estimated by the following EM algorithm.

First let Ψ = {*π*_*k*_, *q*_*kd*_; *k* = 1,…, *K*; *d* = 1,…, *D*} denote all model parameters. The complete data log-likelihood of the parameters can be derived as follows (details are provided in the Supplementary Material available online at http://dx.doi.org/10.1155/2013/617545):

(1) L(Ψ) =∑i∑kδ(Zi=k){log⁡πk+∑d[Yidlog⁡qkd+(1−Yid)                                  ×log⁡(1−qkd)]}.

Here *δ*(·) is an indicator function. Let **Y** denote all observed data, and define *μ*_*ik*_ = *δ*(*Z*_*i*_ = *k*). The *E*-step calculates the expected value of *μ*_*ik*_ given the observed data and the parameter values at the current step, denoted by Ψ^(*t*)^:

(2) μik(t)≡E[δ(Zi=k) ∣ Y,Ψ(t)]=Pr(Zi=k ∣ Y,Ψ(t))=πk(t)P(Yi ∣ Zi=k,Ψ(t))∑k′=1Kπk′(t)P(Yi ∣ Zi=k′,Ψ(t)).

Plugging the expected values into (1), one obtains the Q function as follows:

(3) Q(Ψ ∣ Ψ(t)) =E[l(Ψ) ∣ Y,Ψ(t)] =∑i∑kμik(t){log⁡πk+∑d[Yijlog⁡qkj+(1−Yij)                    ×log⁡(1−qkj)]}.

The *M*-step maximizes the *Q* function with respect to parameters. By solving ∂*Q*/∂*π*_*k*_ = 0 and ∂*Q*/∂*q*_*kd*_ = 0, we obtain the update for *π*_*k*_ and *q*_*kd*_ as follows:

(4) $$\begin{array}{c} \pi_{k}^{\left(t+1\right)}=\frac{\sum_{i}^{}\mu_{ik}^{\left(t\right)}}{I},\;\;q_{kd}^{\left(t+1\right)}=\frac{\sum_{i}^{}\mu_{ik}^{\left(t\right)}Y_{ij}}{\sum_{i}^{}\mu_{ik}^{\left(t\right)}}. \end{array}$$

Because the EM algorithm can sometimes converge to a local maxima, using good starting values is very important. In practice, we choose Ψ^0^ based on *K-*means clustering results. To be specific, we first run *K*-means clustering on **Y** for 10 times and then take the one with the smallest total within cluster distances. The cluster centers and cluster sizes are used as starting values for Ψ. The EM algorithm then iterates between (2) and (4) until convergence.

### 2.3. Choosing the Number of Clusters

 The above EM algorithm is derived with the number of clusters *K* given. Choosing *K* is a model selection problem. A widely used method for obtaining the optimal *K* is the Bayesian Information Criterion (BIC) [11], which is defined as BIC_*K*_ = −2log⁡*L*_*K*_ + *C*_*K*_∗log⁡*T*. Here *L*_*K*_ is the likelihood from the model with *K* clusters, computed based on (1). *C*_*K*_ is the number of parameters, which equals *K*∗(*D* + 1) − 1 in a model with *K* clusters. *T* is the total number of data points, which is *N*∗*D*. The BIC is computed for different values of *K*, and then the *K* associated with the smallest BIC is deemed the to be optimal solution.

 The BIC works well in simulation settings. However in practice, we found that the BIC criteria often favor bigger models. This is a fairly common problem in model selection for genomic data. Because the sample sizes (in this problem, *N*) are often huge, the penalty in BIC is not strong enough to offset the gain in likelihood from bigger models even when effect sizes are small. As a result, BIC often selects a bigger model. In real data analysis, a smaller model is more desirable for interpretability. There are some methods proposed to generate smaller model for genomic data analysis, for example, based on model stability [12] or pruned the larger model down [13]. In this work, we take an easy approach and adopt the recommendation in [14] for choosing number of components in *K*-means clustering. We plot the log-likelihood versus *K* and choose the *K* at the “elbow” point of the curve as the optimal solution. We will show that this *ad hoc* method provides good results in practice.

### 2.4. Interpreting the Model Parameters

 The model parameters are directly interpretable: *π*_*k*_ represents cluster sizes and *q*_*kd*_ represents the probability of occurrence of track *d* in regions of interest from cluster *k*. Under the model assumptions, the parameters sufficiently describe the cooccurrence relationships among input tracks. Any joint or conditional occurrence probabilities can be directly computed from the model parameters. The sufficiency can be shown in the following simple example. The joint probability Pr(*Y*_*ia*_ = 1, *Y*_*ib*_ = 0) for all 1 ≤ *a*, *b* ≤ *D* can be computed as follows:

(5) Pr(Yia=1, Yib=0)  =∑k=1KPr(Yia=1, Yib=0 ∣ Zi=k)Pr(Zi=k)  =∑k=1KPr(Yia=1 ∣ Zi=k)Pr(Yib=0 ∣ Zi=k)πk  =∑k=1Kqka(1−qkb)πk.

Other joint probabilities can be derived in a similar way. The conditional probabilities can be computed from the ratios of proper joint probabilities. These joint/conditional probabilities answer questions like “what is the probability of cobindings of protein A and B?” or “what is the probability of protein A binding at a region, given that protein B binds at that region?” The answers to these questions can be derived from the model parameters without the raw data (e.g., the **Y** matrix). This demonstrates an added advantage of the method: the parameters work like sufficient statistics for cooccurrence patterns among input tracks.

### 2.5. Implementation

 The proposed method has been implemented in an R package giClust, which is freely available at http://www.sph.emory.edu/~hwu/giClust.html. The package takes multiple lists of genomic intervals in BED format as inputs. With two lines of R code the package can generate the results, which include the estimated parameters and the best group assignment for each region of interest.

## 3. Results

 We conducted simulations and real data studies to illustrate the usefulness of giClust. Here we only present the results from two real data tests. The results of two simulation studies can be found in the Supplementary Material.

### 3.1. The Cobinding Patterns of 15 Proteins in Mouse ES Data

 In a seminal paper, Chen et al. mapped the binding sites of 15 different proteins (13 transcription factors and 2 transcription regulators) to study the transcriptional network in mouse embryonic stem (ES) cells [3]. In this example, giClust was applied to discover the cobinding pattern of these proteins. The data were obtained from Gene Expression Omnibus (GEO) database [15] under accession number GSE11431. We first took a union of all called peaks and then used the union as the regions of interest to study the cobinding patterns of all proteins. The union contains 168,439 intervals, with mean length of 542 base pairs. We counted the overlaps between binding sites of all proteins and the unions to construct the **Y** matrix then applied giClust on **Y**, allowing the number of clusters (*K*) to vary from 1 to 15.

**Figure 1:**
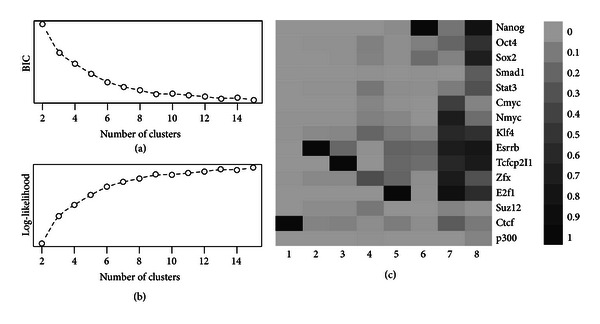
Model fitting results of mouse ES data. (a) BIC versus number of clusters. (b) Log-likelihood versus number of clusters. (c) Estimated $\hat{Q}$ represented as heatmap.

 Figures 1(a)-1(b) plot the BIC and log-likelihood versus *K*. It shows that the BIC kept decreasing with *K* so one cannot obtain an optimal *K* from that. However, looking at the log-likelihood, the amount of increments with *K* is initially large but becomes smaller with larger *K*. The “elbow” point of the curve in Figure 1(b) is at near *K* = 8, so we decided to use *K* = 8 as the optimal number of clusters. The estimated model parameters are summarized in Table 1. The $\hat{Q}$ matrix is also represented as a heatmap in Figure 1(c). 

It shows that a large proportion of the regions of interest are dominated by a single protein; for example, clusters 1, 2, 3, 5, and 6 are almost exclusively bound by CTCF, Esrrb, Tcfcp2l1, E2f1, and Nanog, respectively. Cluster 4 has sparse binding for a number of proteins. Clusters 7 and 8 are the most interesting groups, which are termed as Multiple Transcription Factor-Binding Loci (MTL) in [3]. Cluster 7 (occupies 7% of the regions of interest) is the Myc specific cluster, which shows strong binding tendency from c-Myc, n-Myc, along with several other proteins including Klf4, Esrrb, Tcfcp2l1, Zfx, and E2f1. Cluster 8 (occupies 3% of the regions of interest) is the Nanog-Oct4-Sox2 specific cluster that is defined as “ES-Cell Enhanceosomes” in [3]. This cluster includes strong binding from Klf4, Esrrb, Tcfcp2l1, and E2f1. Moreover 84% of all P300 binding sites belong to this cluster.

**Figure 2:**
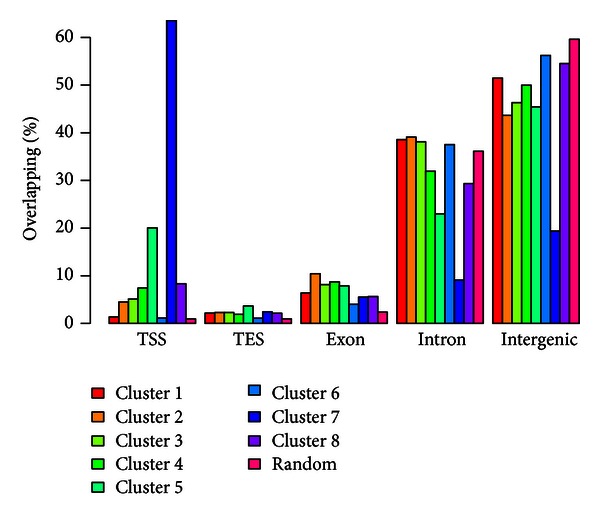
Percentage of genomic regions in each cluster overlapping certain genomic tracks. For example, 63% of the regions in cluster 7 overlap TSS, whereas the expected percentage is only 1%.

We further looked at the locational distributions of regions of interest in different clusters.  Figure 2 shows the percentage of regions in each cluster overlapping some predefined genomic regions, including transcriptional starting site (TSS), transcriptional end site (TES), and exonic/intronic/intergenic regions. The percentages are compared with the expected values (marked as “Random” in Figure 2). The expected values are computed based on total lengths of the predefined genomic features. For example, the total length of TSS regions (defined as ±500 bps around the transcriptional starting location) equals 1% of the total genome. So the expected percentage of genomic regions overlapping TSS is roughly 1%. Note that the percentages for each cluster should sum up to 1. One striking result is that over 60% of intervals in cluster 7 (the Myc specific cluster) overlap TSS, suggesting the regulatory role of Myc proteins in mouse ES cells.

All the findings are consistent with the results reported in [3]. These demonstrate that giClust can discover the cobinding patterns of relatively large number of proteins in a quick analysis and provide biologically meaningful results.

### 3.2. The Histone Modification Pattern over the Gene Promoters in K562 Cell Line

 It was well known that the combinatory patterns of histone modifications correlate with gene expressions. In this example, we used giClust to analyze a number of histone modification datasets. Nine ChIP-seq datasets for profiling different histone modifications in K562 cell lines were obtained from ENCODE. The histones include H3k4me1, H3k4me2, H3k4me3, H3k9me1, H4k20me1, H3k27me3, H3k36me3, H3k9ac, and H3k27ac. In this example, we focused the analysis on the genes and used the promoter regions of Refseq genes as regions of interest.

 Similar to the previous example, we first obtained the overlapping matrix **Y** and then ran giClust for different number of clusters (*K* = 1,…, 10). Plots of the BIC and log-likelihood versus number of clusters are shown in Supplemental Figure S3(a)-(b). The appearance of the curves is similar to that in the mouse ES data. Using a similar method, we picked *K* = 5 as the optimal number of clusters to perform further analyses. The estimated model parameters are listed in Table 2. The heatmap representation of $\hat{Q}$ is shown in Supplemental Figure S3(c).

**Figure 3:**
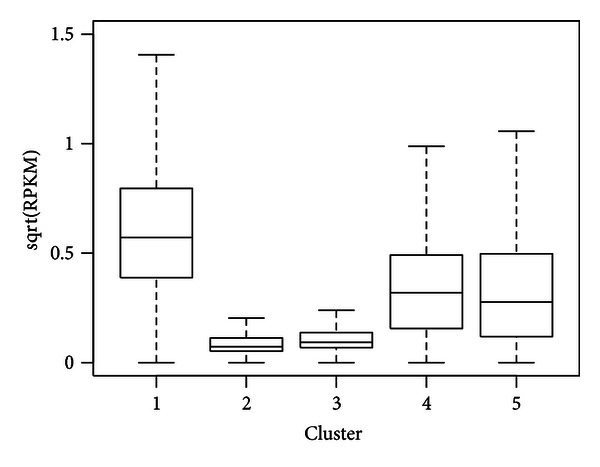
Expressions of genes in different clusters for K562 cell line.

We further compared the expressions of the genes in different clusters. We obtained the RNA-seq data from ENCODE and computed RPKM (reads per kilo-bp per million reads) to represent the gene expressions.  Figure 3 shows the boxplot of expressions, represented as square root of RPKM, for genes in different clusters. It shows that genes in the first cluster (44% of all genes) have the highest average expressions. These genes have modifications on almost all histone marks at promoters except the repressive mark H3K27me3. The second cluster contains 21% genes and shows almost no histone modifications. The expressions for these genes are very low. The third cluster is very interesting. Genes in this cluster have strong modifications on both repressive mark (H3K27me3) and marks associated with activation (H3K9me1 and H4K20me1), yet the gene expressions are low for this cluster. The promoters for these genes probably correspond to the “poised” state as defined in [13]. The fourth cluster (11% of all genes) shows strong enrichment of activation marks, so the gene expressions are relatively high. The difference between the first and fourth clusters is that the genes in the fourth cluster lack the H3k9me1 and H4k20me1 modifications. Although these genes are mostly active, their expressions are on average lower than those in cluster 1. This is possibly due to the additive effects of different activating marks. The fifth cluster (8% of all genes) is enriched with both elongation (H3K36me3) and active marks (H3k9me1) but depleted of the canonical activating marks (H3k4me, H3k9ac, and H3k27ac). The expressions for these genes are relatively high. This potentially suggests alternative mechanisms for gene activation.

In general, the results from this example agree with existing knowledge of the effects of histone modification on gene expression. Yet, they provide some new biological findings worth further exploring. This example further illustrates that a quick analysis by giClust can provide biologically meaningful results.

## 4. Conclusions

With the increasing popularity of ChIP-seq technology, a large amount of data in the form of genomic intervals is generated to represent protein binding or histone modification regions under different biological contexts. Up to date, there has not been an intuitive method available to assess the spatial relationships in these data. To meet this need, we have proposed a method and developed a software tool called “giClust” to characterize the cooccurrence pattern of multiple sets of genomic intervals. The method is based on a finite mixture model. It clusters the regions of interest into several groups according to the cooccurrence patterns of inputs. The model parameters sufficiently capture the overlapping structures in input data. Simulation results showed the method accurately estimates the patterns. Two real datasets showed that the proposed method provides biologically meaningful results. Because there are no existing method or software serving the same purpose, comparisons cannot be performed.

 It is important to distinguish giClust from ChromHMM [7, 13], although they share some similarities. The goal of ChromHMM is to segment the genome according to the combinatory patterns of multiple tracks (in their case, histone modifications), whereas giClust aims to explore the correlation of different tracks. ChromHMM, based on a hidden Markov model (HMM), considers the transitions between consecutive genomic windows, whereas giClust assumes that the regions of interest are independent. The only similarity is the way to model the joint likelihood of multiple tracks of each genomic region, where both methods use product of Bernoulli distributions. Overall giClust and ChromHMM solve different problems and have no dependence on each other.

 The proposed method is designed to work for genomic intervals instead of raw sequencing counts. As discussed in [13], converting the raw count data to presence/absence of peaks before clustering reduces the parameter space and produces more stable and interpretable results. It is also much more computationally efficient since the peak calling results (in the form of genomic intervals) are several orders of magnitude smaller than the raw ChIP-seq sequencing files. Moreover, because different ChIP-seq experiments have distinct technical specifications, clustering directly on raw counts tends to be influenced more by the ones with higher signal to noise ratio. Nevertheless, the method can be extended to include the distribution of raw read counts data in a hierarchical model similar to that one in [16]. This is future research topic worth exploring.

 Choosing the number of clusters *K* is an important step in the analysis. Traditional model selection methods such as BIC tend to favor larger models, which is often difficult to interpret. A software like ChromHMM requires that the user specify number of states. Similarly, we recommend an *ad hoc* procedure to choose *K* from the log-likelihood versus number of cluster curves. We have shown in simulation studies that such a method works reasonably well and is able to capture the major cluster component. However it is still subjective and relies on user input. Automating the process of choosing *K* is an important research topic for future works.

 We use a finite mixture model in this study. An alternative method that we plan to explore is the infinite mixture model, such as a nonparametric Bayesian approach, which has been used previously in genomics research [17].

 The proposed method has been implemented in an easy-to-use R package giClust. We expect the method and the software tool to provide an easy way for biologists to explore their ChIP-seq results and compare with public datasets.

## Supplementary Material

Figure S1: BIC versus number of clusters in simulation data.
Figure S2: BIC and log-likelihood versus number of clusters in a simulation study. The true number of clusters is 8.
Figure S3: Model fitting results of K562 histone modification data. (a) BIC versus number of clusters. (b) Log-likelihood versus number of clusters. (c) Estimated $\hat{Q}$ represented as heatmap.

## Figures and Tables

**Table 1:** The estimated model parameters from mouse ES data with 8 clusters. The $\hat{\pi }$ row shows the estimated cluster sizes. The rest of the table shows $\hat{Q}$, the probability of occurrence of protein binding in different clusters.

Cluster	1	2	3	4	5	6	7	8
$\hat{\pi }$	0.23	0.23	0.18	0.12	0.08	0.07	0.07	0.03

Nanog	0.00	0.00	0.00	0.00	0.03	1.00	0.13	0.85
Oct4	0.00	0.01	0.01	0.07	0.02	0.08	0.20	0.53
Sox2	0.00	0.01	0.01	0.07	0.02	0.17	0.07	0.69
Smad1	0.00	0.00	0.00	0.01	0.00	0.01	0.00	0.25
Stat3	0.00	0.01	0.01	0.12	0.02	0.02	0.10	0.31
Cmyc	0.00	0.00	0.00	0.05	0.01	0.00	0.43	0.06
Nmyc	0.01	0.01	0.01	0.08	0.05	0.00	0.71	0.16
Klf4	0.01	0.04	0.05	0.20	0.11	0.06	0.60	0.53
Esrrb	0.00	1.00	0.19	0.01	0.21	0.20	0.71	0.78
Tcfcp2I1	0.00	0.00	1.00	0.00	0.19	0.16	0.60	0.73
Zfx	0.01	0.04	0.04	0.34	0.21	0.03	0.73	0.31
E2f1	0.00	0.00	0.00	0.00	1.00	0.02	0.86	0.56
Suz12	0.00	0.03	0.03	0.12	0.01	0.01	0.06	0.02
Ctcf	1.00	0.06	0.08	0.02	0.10	0.03	0.25	0.11
p300	0.00	0.00	0.00	0.01	0.00	0.00	0.00	0.06

**Table 2:** The estimated model parameters from K562 histone data with 5 clusters.

Cluster	1	2	3	4	5
$\hat{\pi }$	0.44	0.21	0.16	0.11	0.08

H3k27ac	0.95	0.00	0.01	0.74	0.09
H3k27me3	0.33	0.33	1.00	0.33	0.27
H3k36me3	0.88	0.05	0.02	0.45	0.85
H3k4me1	0.93	0.03	0.20	0.63	0.42
H3k4me2	1.00	0.03	0.18	0.99	0.07
H3k4me3	0.99	0.00	0.12	0.97	0.02
H3k9ac	1.00	0.00	0.02	0.89	0.13
H3k9me1	0.98	0.03	0.79	0.20	0.96
H4k20me1	0.87	0.02	0.87	0.17	0.74

## References

[1] Bird AP (1986). CpG-rich islands and the function of DNA methylation. *Nature*.

[2] Johnson DS, Mortazavi A, Myers RM, Wold B (2007). Genome-wide mapping of in vivo protein-DNA interactions. *Science*.

[3] Chen X, Xu H, Yuan P (2008). Integration of external signaling pathways with the core transcriptional network in embryonic stem cells. *Cell*.

[4] Favorov A, Mularoni L, Cope L (2012). Exploring massive, genome scale datasets with the genometricorr package. *PLoS Computational Biology*.

[5] Chikina M, Troyanskaya O (2012). An effective statistical evaluation of ChIPseq dataset similarity. *Bioinformatics*.

[6] Fu AQ, Adryan B (2009). Scoring overlapping and adjacent signals from genome-wide ChIP and DamID assays. *Molecular BioSystems*.

[7] Ernst J, Kellis M (2012). ChromHMM: automating chromatin-state discovery and
characterization. *Nature Methods*.

[8] Zeng X, Sanalkumar R, Bresnick E, Li H, Change Q, Keles S (2013). jMOSAiCS: joint analysis of multiple ChIP-seq datasets. *Genome Biology*.

[9] Doi A, Park IH, Wen B (2009). Differential methylation of tissue-and cancer-specific CpG island shores distinguishes human induced pluripotent stem cells, embryonic stem cells and fibroblasts. *Nature Genetics*.

[10] Dempster A, Laird N, Rubin D (1977). Maximum likelihood from incomplete data via the EM algorithm. *Journal of the Royal Statistical Society Series B*.

[11] Schwarz G (1978). Estimating the dimension of a model. *The Annals of Statistics*.

[12] Giancarlo R, Utro F (2012). Stability-based model selection for high throughput genomic data: an algorithmic paradigm. *Artificial Immune Systems*.

[13] Ernst J, Kellis M (2010). Discovery and characterization of chromatin states for systematic annotation of the human genome. *Nature Biotechnology*.

[14] Hastie T, Tibshirani R, Friedman J, Franklin J (2005). The elements of statistical learning:
data mining, inference and prediction. *The Mathematical Intelligencer*.

[15] Barrett T, Troup DB, Wilhite SE (2009). NCBI GEO: archive for high-throughput functional genomic data. *Nucleic Acids Research*.

[16] Wu H, Ji H (2010). JAMIE: joint analysis of multiple ChIP-chip experiments. *Bioinformatics*.

[17] Qin ZS (2006). Clustering microarray gene expression data using weighted Chinese restaurant process. *Bioinformatics*.

