# Supplementary material for: Exploring the Cooccurrence Patterns of Multiple Sets of Genomic Intervals
Source: Biomed Res Int. 2013 May 28;2013:617545. doi: 10.1155/2013/617545 (PMC3679813; doi:10.1155/2013/617545)
Supplement: Supplementary file 1 — Figure S1: BIC versus number of clusters in simulation data. Figure S2: BIC and log-likelihood versus number of clusters in a simulation study. The true number of clusters is 8. Figure S3: Model fitting results of K562 histone modification data. (a) BIC versus number of clusters. (b) Log-likelihood versus number of clusters. (c) Estimated Q^ represented as heatmap. [file 617545.f1.pdf]

Appendix

1 Derivation of the complete data log-likelihood

Following the notations in the manuscript, the conditional joint likelihood for observed data at the ith region of interest is:

$$P(\mathbf{Y}_i|Z_i = k) = \prod_{d=1}^{D} [q_{kd}^{Y_{id}}(1 - q_{kd})^{1-Y_{id}}]$$

Here $\mathbf{Y}_i = \{Y_{id}; d = 1, \ldots, D\}$ denotes all data at region of interest i. The joint likelihood of observed and missing data at region of interest i is:

$$P(\mathbf{Y}_i, Z_i) = \prod_{k} \left\{ \pi_k \prod_{d=1}^{D} [q_{kd}^{Y_{id}}(1 - q_{kd})^{1-Y_{id}}] \right\}^{\delta(Z_i=k)}$$

Here $\delta(.)$ is the indicator function. Let $\mathbf{Y} = \{\mathbf{Y}_i; i = 1, \ldots, N\}$, and $\mathbf{Z} = \{Z_i; i = 1, \ldots, N\}$. The whole data likelihood can be expressed as

$$P(\mathbf{Y}, \mathbf{Z}) = \prod_{i} \prod_{k} \left\{ \pi_k \prod_{d} [q_{kd}^{Y_{id}}(1 - q_{kd})^{1-Y_{id}}] \right\}^{\delta(Z_i=k)}$$

Putting these together, the complete data log-likelihood for parameters is:

$$L(\mathbf{q}, \pi) = \sum_{i} \sum_{k} \delta(Z_i = k) \left\{ \log \pi_k + \sum_{d} [Y_{id} \log q_{kd} + (1 - Y_{id}) \log(1 - q_{kd})] \right\}$$

2 Simulation study

2.1 A simple simulation with 3 clusters

We first conducted a simulation study to illustrate the the ideas and validate the estimation procedures of the proposed method. Simulated data were generated for 10,000 regions of interest ($N = 10,000$) and 6 input datasets ($D = 6$). We assumed that there were 3 clusters ($K = 3$), with $\pi = [0.6, 0.3, 0.1]$. The overlapping probability matrix was set to be:

$$\mathbf{Q} = \begin{bmatrix} 0.90 & 0.80 & 0.10 & 0.02 & 0.02 & 0.02 \\ 0.20 & 0.10 & 0.20 & 0.80 & 0.70 & 0.90 \\ 0.90 & 0.80 & 0.70 & 0.80 & 0.70 & 0.80 \end{bmatrix}$$

With these settings, the overlapping matrix $\mathbf{Y}$ was generated based on the mixture model. We then applied `giClust` to determine the optimal number of cluster and estimate model parameters. Figure S1 plots the BIC versus the number of clusters. This showed that the optimal number of clusters was corrected determined as 3.

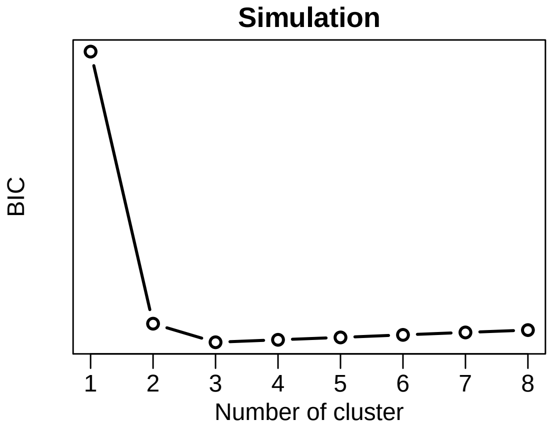

Figure S1: BIC versus number of clusters in simulation data.

Given $K = 3$, the parameter estimations are: $\hat{\pi} = [0.59, 0.30, 0.09]$, and

$$\hat{\mathbf{Q}} = \begin{bmatrix} 0.91 & 0.81 & 0.10 & 0.02 & 0.02 & 0.02 \\ 0.21 & 0.08 & 0.20 & 0.81 & 0.72 & 0.91 \\ 0.88 & 0.85 & 0.69 & 0.78 & 0.69 & 0.80 \end{bmatrix}$$

These show that the estimation procedures work well, e.g., the parameters can be accurately estimated. It further shows that BIC selects the correct number of cluster in this simple case.

2.2 A bigger simulation with 10 clusters

We further performed a bigger simulation that is closer to data from real experiments. In this simulation, there are 10 datasets ($D = 10$), 100,000 regions of interests ($N = 100,000$), and 8 true underlying clusters ($K = 8$). The cluster sizes are $\pi = [0.4, 0.2, 0.2, 0.08, 0.04, 0.03, 0.02, 0.02]$. The overlapping probability matrix $\mathbf{Q}$ used in the simulation is:

This simulation resembles the real data scenario, e.g., there are a few "major" clusters occupying most of the genome, and many other smaller clusters. In this case, for better interpretability of the results, one often wants a smaller model that captures the major clusters. In this simulation, the main aim is to evaluate the *ad hoc* model selection procedure.

	Data1	Data2	Data3	Data4	Data5	Data6	Data7	Data8	Data9	Data10
cluster 1	0.80	0.80	0.80	0.80	0.80	0.20	0.20	0.20	0.20	0.20
cluster 2	0.20	0.20	0.20	0.20	0.20	0.80	0.80	0.80	0.80	0.80
cluster 3	0.80	0.80	0.80	0.80	0.80	0.80	0.80	0.80	0.80	0.80
cluster 4	0.80	0.59	0.91	0.88	0.99	0.90	0.88	0.20	0.33	0.78
cluster 5	0.16	0.08	0.13	0.17	0.48	0.70	0.88	0.88	0.85	0.17
cluster 6	0.52	0.87	0.23	0.12	0.82	0.08	0.95	0.87	0.71	0.61
cluster 7	0.23	0.74	0.90	0.29	0.52	0.43	0.58	0.74	0.68	0.36
cluster 8	0.33	0.75	0.51	0.86	0.76	0.96	0.83	0.84	0.27	0.18

We applied `giClust` on the simulated $\mathbf{Y}$ matrix, and varied number of clusters from 1 to 8. The BIC and log-likelihood plots are shown in Figure S2. The BIC decreases when the number of clusters increases, because the real number of clusters is 8.

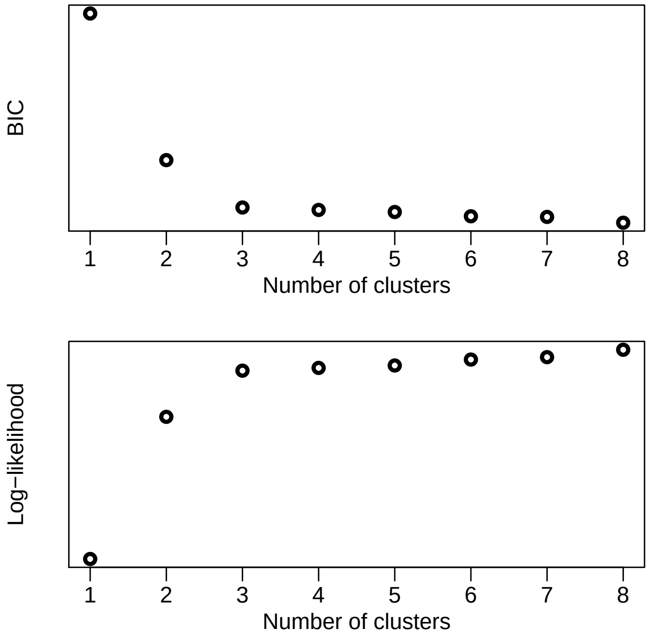

Figure S2: BIC and log-likelihood versus number of clusters in a simulation study. The true number of clusters is 8.

However for interpretability, we picked the elbow point of the log-likelihood plot and fixed the number of clusters as 3. Using `giClust` on the data, we obtained the estimated overlapping probability matrix $\hat{\mathbf{Q}}$ as:

The estimated cluster sizes are $\hat{\pi} = [0.40, 0.32, 0.27]$. One can see that the overlapping prob-

	Data1	Data2	Data3	Data4	Data5	Data6	Data7	Data8	Data9	Data10
Cluster1	0.78	0.80	0.80	0.79	0.80	0.20	0.20	0.21	0.20	0.19
Cluster2	0.77	0.75	0.81	0.80	0.85	0.78	0.81	0.63	0.63	0.74
Cluster3	0.21	0.25	0.21	0.20	0.29	0.73	0.81	0.82	0.80	0.67

abilities are very well estimated for the 3 major clusters. The cluster sizes estimates are a little biased because the smaller clusters were absorbed into the the major ones. Overall the results reasonably represent the overlapping patterns, and the *ad hoc* model selection procedure works fairly well.

3 Results from K562 histone modification example

The figure below summarizes the model fitting results for K562 histone modification data.

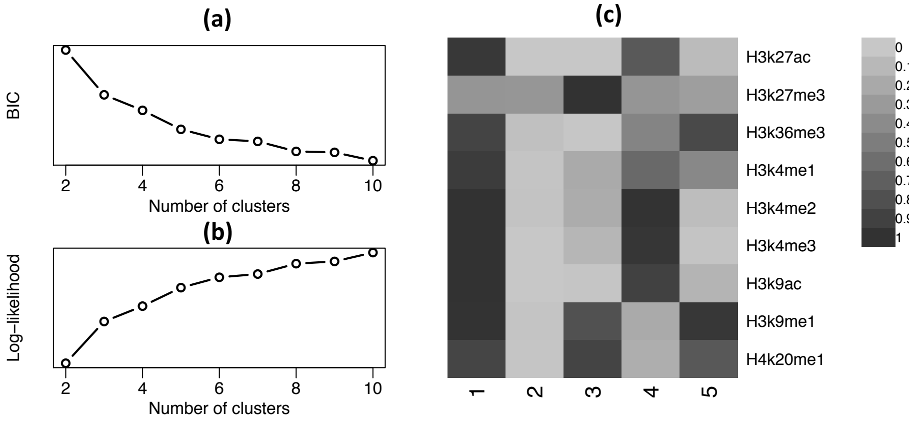

Figure S3: Model fitting results of K562 histone modification data. (a) BIC versus number of clusters. (b) Log-likelihood versus number of clusters. (c) Estimated $\hat{\mathbf{Q}}$ represented as heatmap.
